# Fentanyl Detection in Biological Samples From Individuals Reporting Synthetic Cannabinoid Use in São Paulo, Brazil

**DOI:** 10.1111/dar.70266

**Published:** 2026-09-15

**Authors:** Ligia Goes Endo, Rodrigo Alves de Oliveira, Ivan Dieb Miziara, Mauricio Yonamine, João Maurício Castaldelli‐Maia, Vilma Leyton, Henrique Silva Bombana

**Affiliations:** ^1^ Departamento de Medicina Legal, Bioética, Medicina Do Trabalho e Medicina Física e Reabilitação, Faculdade de Medicina Universidade de São Paulo São Paulo Brazil; ^2^ Departamento de Análises Clínicas e Toxicológicas, Faculdade de Ciências Farmacêuticas Universidade de São Paulo São Paulo Brazil; ^3^ Departamento de Psiquiatria, Faculdade de Medicina Universidade de São Paulo São Paulo Brazil; ^4^ Instituto Perdizes, Hospital das Clínicas HCFMUSP, Faculdade de Medicina, Universidade de São Paulo São Paulo Brazil

**Keywords:** fentanyl, oral fluid, synthetic cannabinoid, toxicological surveillance

## Abstract

**Introduction:**

Brazil has historically exhibited low illicit opioid prevalence, though forensic data indicate rising fentanyl seizures, often in combination with other substances. People who use Synthetic cannabinoid receptor agonists (SCRA) are particularly vulnerable to unintentional fentanyl exposure due to the unpredictable chemical composition of these products. This study aimed to investigate the discordance between self‐reported drug use and toxicological detection of fentanyl among individuals reporting recent SCRA use recruited from addiction treatment and psychosocial care centres in São Paulo.

**Methods:**

Cross‐sectional study with 65 participants reporting SCRA use within the preceding 4 days, recruited from a specialised addiction treatment centre and two community‐based psychosocial care centres in São Paulo. Oral fluid samples collected by independent research staff using the Quantisal device were analysed by liquid chromatography–tandem mass spectrometry. Sociodemographic characteristics, self‐reported substance use and clinical symptoms were assessed by structured questionnaire. Exploratory multivariable logistic regression investigates factors associated with fentanyl detection.

**Results:**

Fentanyl was detected in 32% of participants (21/65; 95% CI 21, 44), none of whom reported intentional opioid use. Fentanyl‐positive participants were more likely to be experiencing homelessness (52.4% vs. 13.6%, *p* = 0.002), to report longer SCRA use duration (*p* = 0.001), and to present with nausea/vomiting (90.5% vs. 43.2%, *p* < 0.001) and myalgia (61.9% vs. 11.4%, *p* < 0.001). No independent factors associated with fentanyl detection were identified in exploratory multivariable analyses.

**Discussion and Conclusions:**

A substantial discordance between self‐reported drug use and toxicological findings was observed. While the small sample size limits generalizations, these findings provide preliminary evidence that the historical assumption of negligible illicit opioid exposure in this population warrants reassessment. Whether this reflects changes in the local drug supply, underreporting of opioid use, or other exposure pathways cannot be determined from the available data. These results underscore the need for systematic toxicological surveillance and drug checking to monitor the evolving drug supply in Brazil.

## 1 Introduction

Brazil has historically exhibited a low prevalence of illicit opioid use compared to North American and European contexts [1]. Non‐medical opioid consumption was traditionally considered rare, even among socially vulnerable populations [2, 3]. However, recent reports from national early warning systems and forensic data have documented a concerning increase in non‐therapeutic fentanyl seizures across the country [4]. Regional experts have warned that while a widespread crisis may still be averted, the rising detection of fentanyl in contemporary Brazil is a significant public health priority [5]. This shifting landscape is further complicated by the recent emergence of other highly potent synthetic opioids, such as nitazenes, which have been detected in seizures in São Paulo and other Brazilian states since 2022 [6].

The unpredictability of the illicit market is underscored by the frequent co‐occurrence of fentanyl with various non‐opioid substances [5]. Recent forensic surveillance data from São Paulo documented fentanyl co‐occurring with Δ9‐tetrahydrocannabinol, synthetic cannabinoid receptor agonists (SCRA), and cocaine in seized samples [7]. In Brazil, SCRAs (locally known as spice, K2, or K9) have been increasingly identified in the drug supply since 2014, often marketed to vulnerable urban populations [8]. SCRA use among marginalized groups is characterized by polypharmacy and unpredictable consumption patterns [9, 10], rendering these individuals particularly vulnerable to unrecognized opioid exposure. Despite robust forensic seizure evidence, toxicologically confirmed fentanyl exposure in biological samples from people who use drugs in Brazil remains absent from the peer‐reviewed literature to our knowledge; confirmed fentanyl consumption has been documented only in isolated cases [4]. Whether this fentanyl co‐occurrence represents genuine co‐use or product contamination remains unclear at the individual level.

Given the potential for unrecognized fentanyl exposure and associated clinical risks, this study investigated the discordance between self‐reported drug use and toxicological detection of fentanyl among individuals reporting recent SCRA use in São Paulo.

## 2 Methods

### 2.1 Setting and Participants

Between August 2024 and August 2025, 65 individuals reporting SCRA use were recruited from a specialized addiction treatment center and two community‐based psychosocial care centers in São Paulo, Brazil. Eligibility criteria included self‐reported SCRA use within the 4 days before assessment. Two potential participants were excluded because they were clinically unable to respond to the assessment or provide informed consent due to drug intoxication. Participation was voluntary and no compensation was provided. The structured questionnaire took approximately 6 min to complete. All individuals who completed the survey provided an oral fluid sample, and no exclusions were made between survey completion and biological testing. The study was approved by the Research Ethics Committee of the Hospital das Clínicas, University of São Paulo Medical School (CAPPesq‐HCFMUSP #76109023.2.0000.0068), and all participants provided written informed consent.

### 2.2 Data and Sample Collection

Participants completed a structured questionnaire assessing sociodemographic characteristics, housing and employment status, patterns of substance use, and self‐reported signs and symptoms related to SCRA use. ‘Patterns of substance use’ referred to frequency of use and the use of other substances (cocaine, cannabis, alcohol, crack cocaine and medicines) within the same four‐day call period. The ‘medicines’ category was defined as prescribed or over‐the‐counter pharmaceutical products (e.g., benzodiazepines, anticonvulsants or non‐opioid analgesics) and did not include illicitly acquired drugs or fentanyl. Given that this study centres on fentanyl detection, self‐reported fentanyl and other opioid use (e.g., heroin, morphine, methadone, codeine and oxycodone) was explicitly assessed through dedicated, structured questions to establish whether participants knowingly or intentionally consumed any opioid product. Acute effects were defined as symptoms experienced during or immediately after the last use, while withdrawal symptoms referred to those experienced during abstinence periods within the preceding 4 days. Oral fluid samples were collected using the Quantisal device for toxicological analysis. Samples were collected on‐site by independent research staff, refrigerated at 4°C, and transferred to the Toxicology Laboratory at FMUSP, where they were maintained at −20°C until analysis.

### 2.3 Toxicological Analysis

Samples were analysed by liquid chromatography–tandem mass spectrometry [11]. The testing panel included fentanyl, SCRAs, cocaine (benzoylecgonine and anhydroecgonine methyl ester—AEME), THC, ketamine and amphetamine‐type stimulants (amphetamine, methamphetamine and MDMA). A detailed list of all target analytes and their respective cut‐offs is provided in Table S1.

### 2.4 Statistical Analysis

Categorical variables were summarized as absolute and relative frequencies. Continuous variables (age) were described using medians and interquartile ranges. Given that local seizure data indicated fentanyl co‐occurrence with SCRA, we examined whether this pattern had clinical correlates by comparing SCRA‐related characteristics across fentanyl detection status using Fisher's exact test and Mann–Whitney U tests. Variables relating to acute effects and withdrawal symptoms were included in the model to account for the possibility that individuals with unrecognized fentanyl co‐exposure may present with a distinct clinical phenotype compared with those using SCRAs alone. Multivariable logistic regression explored factors associated with fentanyl detection, including variables with *p* < 0.20 in bivariate analyses (education, homelessness and duration of use). As the study protocol was not pre‐registered, these findings should be considered exploratory. All statistical analyses were conducted using IBM SPSS Statistics (version 30.0; IBM Corp., Armonk, NY, USA). A two‐tailed *p* < 0.05 was considered statistically significant.

## 3 Results

### 3.1 Sample Characteristics

Participants were predominantly male (83%) and young adults (median age of 23 years and interquartile range 20, 29). Most were single (82%), unemployed (63%) and 68% had not completed secondary education. At the time of the interview, 42% (27/65) were experiencing homelessness (including those in shelters). Regarding SCRA use, 74% reported a duration of use using SCRA for over 3 years. In Brazil, these substances are commonly marketed under street names such as K2, K9, and ‘space’, with K9 being the most frequently reported product in this sample. Participants described two perceived potency variants of K9, locally referred to as ‘strong’ and ‘weak’, with the latter more commonly used due to milder subjective effects. However, these perceived differences in potency were not analytically compared, as no participant reported the exclusive use of the ‘strong’ variant during the assessed period, preventing a direct toxicological correlation.

Use of other substances during the same four‐day reporting period was common, with 23% of participants reporting cocaine use, 20% cannabis, 17% alcohol, and 17% crack cocaine. Overall, 46% of participants reported using at least one substance other than SCRAs during this window.

### 3.2 Toxicological Findings

**FIGURE 1:**
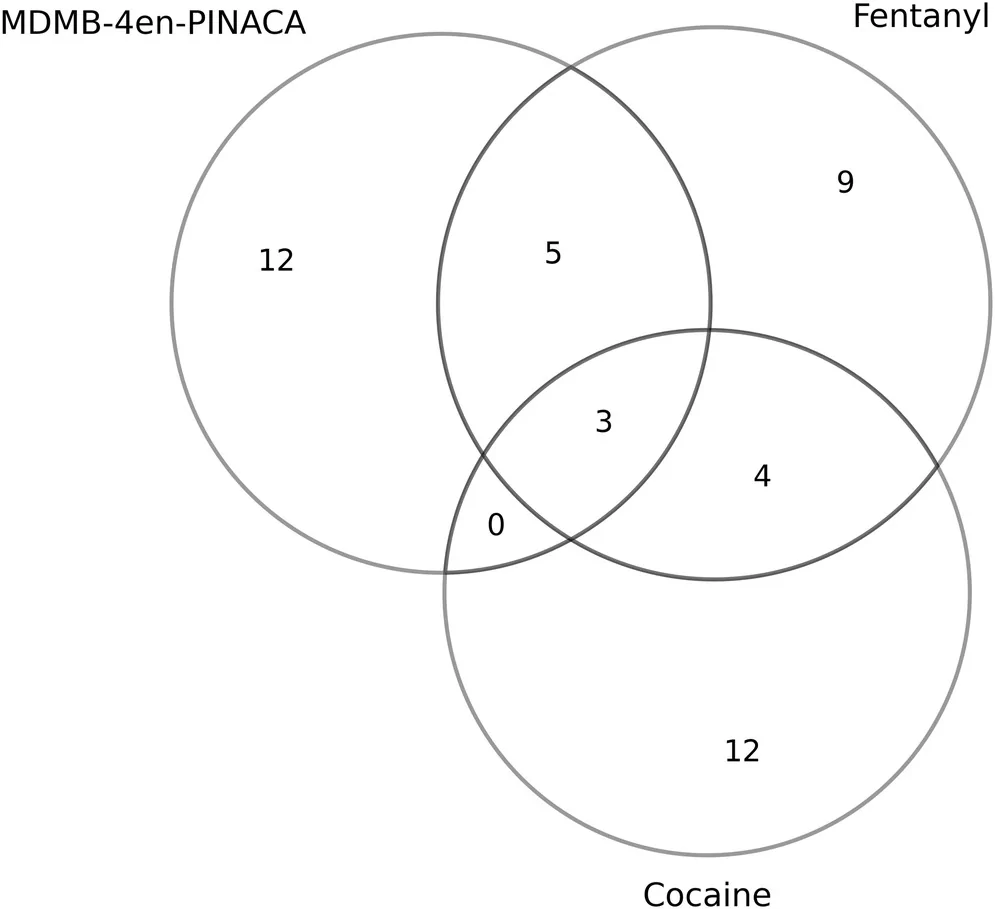
Toxicological co‐detection patterns among participants with confirmed drug exposure (*n* = 45).

Forty‐five samples (69%) tested positive results for at least one substance. Fentanyl was detected in 32% of participants (21/65; 95% confidence interval 21, 44), none of whom reported intentional use of fentanyl or any other opioid drug more broadly. All fentanyl‐positive individuals reported SCRA use within the preceding 48 h, although not all tested positive for SCRAs. This discordance likely reflects analytical limitations in capturing the rapid emergence of novel synthetic cannabinoid compounds for which reference standards are not yet available. Among the 21 participants with detectable fentanyl, 9 tested positive for fentanyl only, 5 tested positive for both fentanyl and MDMB‐4en‐PINACA, 4 tested positive for fentanyl and cocaine/metabolites, and 3 presented with a triple co‐detection of fentanyl, MDMB‐4en‐PINACA, and cocaine/metabolites (Figure 1). None of the samples tested positive for amphetamine‐type stimulants.

Participant characteristics stratified by fentanyl detection status are presented in Table 1. Fentanyl‐positive participants were significantly more likely to be experiencing homelessness and to report longer duration of SCRA use (*p* < 0.01). Paradoxically, this subgroup reported lower rates of concomitant cannabis, cocaine, and alcohol use (all *p* < 0.05). Regarding clinical presentation, nausea/vomiting (91% vs. 43%, *p* < 0.001) and myalgia were markedly more frequent among fentanyl‐positive participants. Multivariable logistic regression identified no independent factors associated with fentanyl detection among the assessed.

**TABLE 1:** Characteristics of participants by fentanyl detection status.

Variable	Fentanyl‐positive (*n* = 21)	Fentanyl‐negative (*n* = 44)	*p* *
Sociodemographic			
Age, median (IQR)	23 (21, 2 5)	25 (21, 31)	0.162
Male sex, *n* (%)	19 (91%)	35 (80%)	0.480
Single, *n* (%)	16 (76%)	34 (77%)	1.000
Education below secondary, *n* (%)	17 (81%)	23 (52%)	**0.032**
Unemployed, *n* (%)	11 (52%)	26 (59%)	0.789
Homeless, *n* (%)	11 (52%)	6 (14%)	**0.002**
Synthetic cannabinoid use pattern			
Duration of SCRA use ≥ 3 years, *n* (%)	18 (86%)	18 (41%)	**0.001**
Self‐reported use of other substances			
Cannabis, *n* (%)	0 (0%)	13 (30%)	**0.006**
Cocaine, *n* (%)	0 (0%)	15 (34%)	**0.001**
Crack cocaine, *n* (%)	2 (10%)	9 (21%)	0.480
Alcohol, *n* (%)	0 (0%)	11 (25%)	**0.012**
Medicines**, *n* (%)	1 (5%)	20 (45%)	**< 0.001**
Acute effects of synthetic cannabinoid use			
Nausea/vomiting, *n* (%)	19 (91%)	19 (43%)	**< 0.001**
Syncope, *n* (%)	11 (52%)	14 (32%)	0.172
Relaxation, *n* (%)	12 (57%)	22 (50%)	0.789
Increased energy, *n* (%)	5 (24%)	3 (7%)	0.100
Withdrawal symptoms			
Myalgia, *n* (%)	13 (62%)	5 (11%)	**< 0.001**
Chills, *n* (%)	7 (33%)	6 (14%)	0.096
Tremors, *n* (%)	5 (24%)	13 (30%)	0.770
Sweating, *n* (%)	3 (14%)	13 (30%)	0.229
Anxiety, *n* (%)	1 (5%)	2 (5%)	1.000

*Note:* Bold *p* indicate *p* < 0.05.

Abbreviations: IQR, interquartile range; SCRA, synthetic cannabinoid receptor agonists.

* *p* from Fisher's exact test (categorical variables) or Mann–Whitney U test (continuous variables).

** Benzodiazepines, anticonvulsants, or non‐opioids analgesics.

## 4 Discussion

The present study identified fentanyl in oral fluid samples from 32% of participants, none of whom reported intentional opioid use. Against a backdrop where illicit opioid use has traditionally been considered rare in Brazil [5], even among socially vulnerable populations, the discordance between self‐reported use and toxicological findings observed in this study aligns with international reports of unexpected fentanyl exposure in unpredictable drug markets [12, 13]. Whether this reflects changes in the local drug supply, underreporting of opioid use or other exposure pathways remains to be determined. However, the detection of fentanyl in biological samples from people who use drugs in Brazil, documented here for the first time to our knowledge, provides preliminary evidence that the historical assumption of negligible illicit opioid exposure in this population warrants reassessment. While the small sample size and specific recruitment settings limit broad generalizations, these findings signal a potentially shifting landscape that requires urgent attention.

Several explanations for this discordance warrant consideration. First, fentanyl may have been present as an adulterant in the SCRA products, a hypothesis consistent with prior forensic data from São Paulo [4, 14]. Second, underreporting of intentional opioid use cannot be excluded. Although surveys were administered by independent research staff rather than treatment personnel to mitigate social desirability bias, the reliability of self‐reported data is challenged by the high prevalence of unintentional consumption in adulterated substances [15]. As many consumers are unaware that they are encountering substances contaminated with highly potent synthetic opioids, collecting comprehensive data through self‐report has become increasingly difficult [1, 6]. Notably, prior research has shown that individuals who use fentanyl‐type drugs are more likely to underreport their use and have limited knowledge of fentanyl analogues present in their drug supply [12]. Third, the finding that fentanyl‐positive participants reported significantly lower rates of concomitant cannabis, cocaine, and alcohol use suggests a more exclusive pattern of SCRA use potentially concentrating exposure within SCRA‐specific supply chains rather than representing adulterants in cocaine‐based products. While seizure data from São Paulo documented fentanyl co‐occurring with cocaine [4, 14], our sample revealed that fentanyl‐positive participants reported significantly lower rates of concomitant cocaine use compared to fentanyl‐negative individuals (0% vs. 34%, *p* = 0.001). Similar patterns of discordance between self‐reported drug use and toxicological findings have been documented in Toronto and the US, where market unpredictability leads to widespread unexpected fentanyl exposure [13, 16]. The convergence of these international observations with local seizure data warrants serious attention to the possibility that the Brazilian drug supply is undergoing similar structural changes.

Regardless of the underlying mechanism, the clinical implications of fentanyl detection in this population warrant serious concern. Fentanyl was present in individuals who did not disclose opioid use, meaning that clinicians cannot rely on self‐reported drug use alone to guide assessment. The combination of fentanyl and SCRAs carries particular pharmacological dangers. Experimental evidence suggests that co‐administration can exacerbate respiratory depression and confer resistance to naloxone rescue [17]. A fatal case of acute mixed intoxication involving fentanyl and SCRA further illustrates the lethal potential of this combination in real‐world use contexts [18]. Clinicians should therefore be aware that standard naloxone protocols may be insufficient in such cases, and higher or repeated doses may be required.

Whether these findings reflect an isolated occurrence or an emerging pattern within the Brazilian illicit drug supply remains unknown. Notably, participants in rural US settings have described fentanyl as having saturated both opioid and stimulant supplies, with most people who use drugs employing multiple simultaneous strategies to reduce overdose risk [19], a harm reduction infrastructure that is currently absent in Brazil. While differences in market dynamics exist, the trajectory of North America's opioid crisis, where early, sporadic fentanyl detections preceded a public health emergency, offers a cautionary precedent for the risks of unmonitored supply adulteration [18]. Establishing continuous drug checking and robust toxicological surveillance now, before fentanyl becomes further entrenched in Brazilian drug markets, represents both an urgent public health priority and a critical opportunity to potentially avoid repeating that trajectory.

### 4.1 Limitations

This study is limited by its small sample size and recruitment from specialized services, which may affect generalizability. Self‐reported substance use may also be subject to underreporting and social desirability bias, particularly regarding stigmatized substances such as opioids. Additionally, the rapid emergence of new compounds and the lack of analytical standards may lead to an underestimation of exposure to specific substances. Furthermore, our testing panel was focused on fentanyl, SCRA and ‘classic’ drugs such as cocaine, cannabis and amphetamine‐type stimulants; therefore, exposure to other emerging synthetic opioids, such as nitazenes, or traditional opioids was not assessed. Importantly, this study did not include analysis of the substances consumed by participants. Therefore, it is not possible to determine whether fentanyl exposure resulted from contamination of SCRA products, unreported opioid use or other exposure pathways.

## 5 Conclusion

The detection of fentanyl among individuals without reported opioid use highlights the need to investigate potential sources, including possible contamination of the drug supply in São Paulo. Whether this represents an isolated finding or reflects a broader phenomenon within the Brazilian illicit drug market remains unclear. Establishing sentinel toxicological surveillance systems is essential to monitor these emerging trends and inform harm reduction responses.

## Author Contributions

**Ligia Goes Endo:** conceptualization, methodology, data curation, writing – original draft; **Rodrigo Alves de Oliveira:** investigation; **Ivan Dieb Miziara:** resources; **Mauricio Yonamine:** writing – review and editing; **João Maurício Castaldelli‐Maia:** writing – review and editing; **Vilma Leyton:** visualization, resources; **Henrique Silva Bombana:** writing – review and editing, supervision, project administration.

## Funding

This study was supported by the Coordination for the Improvement of Higher Education Personnel (CAPES), Brazil (Grant number: 88887.198549/2025–00), HSB received a scholarship from the São Paulo Research Foundation (FAPESP), Brazil. Process Number #2024/04999–7.

## Conflicts of Interest

The authors declare no conflicts of interest.

## Supporting information


**Data S1:** dar70266‐sup‐0001‐Supinfo.docx.

## Data Availability

The data that support the findings of this study are available from the corresponding author upon reasonable request.
