# Supplementary material for: Fentanyl Detection in Biological Samples From Individuals Reporting Synthetic Cannabinoid Use in São Paulo, Brazil
Source: Drug Alcohol Rev. 2026 Sep 15;45(6):e70266. doi: 10.1111/dar.70266 (PMC13579073; doi:10.1111/dar.70266)
Supplement: Supplementary file 1 [file DAR-45-0-s001.docx]

| **Analyte** | **Cutoff (ng/mL)** | **Internal standard** |
| --- | --- | --- |
| 4-cyano MMB-BUTINACA | 1 | THC-D3 |
| ADB-FUBIATA | 2 | THC-D3 |
| ADB-BINACA | 1 | THC-D3 |
| 4-Fluoro MDMB-BUTICA | 1 | THC-D3 |
| 4-Fluoro MDMB-BUTINACA | 1 | THC-D3 |
| 5-Fluoro EDMB-PICA | 1 | THC-D3 |
| MDMB-4en-PINACA | 1 | THC-D3 |
| JWH-073 | 1 | THC-D3 |
| 4-Fluoro-ABUTINACA | 1 | THC-D3 |
| BZO-CHMOXIZID | 1 | THC-D3 |
| Δ9-tetrahydrocannabinol | 10 | THC-D3 |
| Cocaine | 10 | Benzoylecgonine-D3 |
| Benzoylecgonine | 10 | Benzoylecgonine-D3 |
| Anydro ecgonine methyl esther | 10 | Benzoylecgonine-D3 |
| Fentanyl | 1 | Fentanyl-D5 |
| Ketamine | 1 | Ketamine-D4 |
| Amphetamine | 10 | MDMA-D5 |
| Methamphetamine | 10 | MDMA-D5 |
| MDMA | 10 | MDMA-D5 |
